# α5‐nAChR contributes to epithelial‐mesenchymal transition and metastasis by regulating Jab1/Csn5 signalling in lung cancer

**DOI:** 10.1111/jcmm.14941

**Published:** 2020-01-13

**Authors:** Xiaowei Chen, Yanfei Jia, Yujie Zhang, Dajie Zhou, Haiji Sun, Xiaoli Ma

**Affiliations:** ^1^ Central Laboratory Jinan Central Hospital Affiliated to Shandong University Jinan China; ^2^ Weifang Medical University Weifang China; ^3^ Key Laboratory of Animal Resistance Biology of Shandong Province School of Life Science Shandong Normal University Jinan China

**Keywords:** epithelial‐mesenchymal transition, Jab1/Csn5, non‐small‐cell lung cancer, α5‐nicotinic acetylcholine receptor

## Abstract

Recent studies have showed that α5 nicotinic acetylcholine receptor (α5‐nAChR) is closely associated with nicotine‐related lung cancer. Our previous studies also demonstrated that α5‐nAChR mediates nicotine‐induced lung carcinogenesis. However, the mechanism by which α5‐nAChR functions in lung carcinogenesis remains to be elucidated. Jab1/Csn5 is a key regulatory factor in smoking‐induced lung cancer. In this study, we explored the underlying mechanisms linking the α5‐nAChR‐Jab1/Csn5 axis with lung cancer epithelial‐mesenchymal transition (EMT) and metastasis, which may provide potential therapeutic targets for future lung cancer treatments. Our results demonstrated that the expression of α5‐nAChR was correlated with the expression of Jab1/Csn5 in lung cancer tissues and lung cancer cells. α5‐nAChR expression is associated with Jab1/Csn5 expression in lung tumour xenografts in mice. In vitro, the expression of α5‐nAChR mediated Stat3 and Jab1/Csn5 expression, significantly regulating the expression of the EMT markers, N‐cadherin and Vimentin. In addition, the down‐regulation of α5‐nAChR or/and Stat3 reduced Jab1/Csn5 expression, while the silencing of α5‐nAChR or Jab1/Csn5 inhibited the migration and invasion of NSCLC cells. Mechanistically, α5‐nAChR contributes to EMT and metastasis by regulating Stat3‐Jab1/Csn5 signalling in NSCLC, suggesting that α5‐nAChR may be a potential target in NSCLC diagnosis and immunotherapy.

## 1 INTRODUCTION

Lung cancer is the most prevalent carcinoma and the leading cause of cancer death worldwide. Approximately 85%‐90% of lung cancer diagnoses are non‐small‐cell lung cancer (NSCLC), including lung adenocarcinoma (LUAD) and squamous cell carcinoma (LUSC). A remarkable proportion of cases are only detected in the advanced stage when the tumour has already metastasized to other organs, resulting in poor clinical prognosis.^1^, ^2^ Epithelial‐mesenchymal transition (EMT) is generally considered to be the key progression promoting the metastasis of lung cancer.^3^ Thus, elucidating the molecular mechanism of EMT is of great significance in preventing lung cancer metastasis and progression.

Nicotinic acetylcholine receptors (nAChRs) are widely distributed in various types of cancer cells, including lung, breast, pancreas, stomach and gliomas, and regions where there are ligand‐gated ion channels.^4^ Nicotinic acetylcholine receptors comprise pentameric subunits using various combinations of alpha (α1‐α10) or non‐alpha (β1‐β4, γ, δ, or ε) types.^5^ Nicotinic acetylcholine receptors are classified into two categories, heteromeric and homomeric nAchRs.^6^ Heteromeric nAChRs contain a combination of α and β subunits. Homomeric nAChRs are composed of five α subunits (α7, α8 and α9).^7^ Previous studies have demonstrated that nAChRs play an important role in mediating the stimulation of tumour cell proliferation, migration, invasion and EMT of lung cancer.^8^, ^9^ Different subunits of nAChRs are expressed in different tissues and organs.^10^, ^11^ Among different members of nicotinic receptor family, the intracellular domain of α7 nAChR is one of the most well conserved, and the role of α7‐nAChR in non‐neuronal systems has been predominantly studied.^12^ However, nAChR subunits and downstream signalling pathways vary from different types of NSCLC.^13^ Notably, genome‐wide association studies (GWAS) have showed that variants in the region encoding the α3, β4 and α5 subunits of nAChRs are significantly involved in nicotine dependence and lung carcinogenesis.^14^ Especially, α5‐nAChR was highly associated with lung cancer risk and nicotine dependence.^15^, ^16^ Here, we have focused on the role of α5‐nAChR in the development and progression of lung cancer.

Recently, our laboratory demonstrated that α5‐nAChR mediates nicotine‐induced lung cancer development and progression.^17^ Nicotine interacts with α5‐nAChR on the cell surface, activating the JAK2/Stat3 signalling pathways and promoting lung cancer cell proliferation.^18^ Furthermore, α5‐nAChR mediates nicotine‐induced lung cancer cell migration and invasion.^19^ Nevertheless, the mechanism by which α5‐nAChR functions in lung cancer EMT remains to be elucidated.

Jab1 was originally identified as a c‐Jun coactivator and was subsequently shown to be the fifth member of the constitutive photomorphogenic‐9 (COP9) signalosome (CSN) complex (COPS5 or Csn5, commonly known as Jab1; Jab1 hereafter).^20^ Increasing evidence indicates that Jab1 activity mediates various tumorigenesis‐associated pathways^21^ and is a potential target for smoking‐induced lung cancer.^22^ P‐Stat3 regulates the progression of nasopharyngeal and breast cancer by activating Jab1,^23^, ^24^ which regulates the expression of ZEB1 and affects the EMT process in renal cancer cells (RCCs).^25^ Notably, the down‐regulation of Jab1 decreases PD‐L1 expression in cancer cells and sensitizes them to anti‐CTLA4 therapy.^26^, ^27^ Thus, understanding the regulation of α5‐nAChR and Jab1 expression has major clinical relevance.

In this study, we assessed α5‐nAChR and Jab1 expression in LUAD and demonstrated that Jab1 expression is positively associated with α5‐nAChR levels in vivo. Furthermore, α5‐nAChR mediates the EMT and metastasis of NSCLC cells via Stat3/Jab1 signalling in LUAD. To the best of our knowledge, this is the first study to show that the α5‐nAChR/Jab1 signalling axis is involved in lung cancer EMT and metastasis, which may represent a plausible tumour‐targeting strategy in lung cancer.

## 2 MATERIALS AND METHODS

### 2.1 Tissue specimens and cell cultures

A tissue microarray (No.HLug‐Ade050CD‐01; Xinchao Biotechnology) containing 18 adenocarcinoma specimens and 18 para‐carcinoma tissues was used in this study. Each set of paired tumour and para‐carcinoma tissues was collected and categorized according to their clinical information. Among the 18 samples, eight were collected from males and 10 were collected from females, with an overall age range of 45‐75 years (age average, 59.6 years).

The human NSCLC cell lines A549 and H1299 were purchased from the Cell Resource Center of the Chinese Academy of Sciences (China). Cells were cultured in RPMI‐1640 medium (HyClone) supplemented with 10% foetal bovine serum (FBS; HyClone) at 37°C in a humidified atmosphere of 5% CO_2_ in air.

### 2.2 Clinical cDNA microarray analysis of the correlations between CHRNA5 and Jab1 (COSP5) using relevant databases

The expression patterns of α5‐nAChR and Jab1 (COSP5) in TCGA LUAD (n = 515) and normal tissues (n = 59) were analysed using the Ualcan online database (http://ualcan.path.uab.edu/index.html). The correction between the expression of CHRNA5 and Jab1 (COSP5) in NSCLC was analysed using the R2 online database (http://r2.amc.nl). The correlation between CHRNA5 and/or Jab1 (COSP5) expression and the cancer patients' overall survival in TCGA LUAD dataset was analysed using R2: Kaplan Meier Scanner (Pro) (https://hgserver1.amc.nl/cgi-bin/r2/main.cgi).

### 2.3 In vivo proliferation and metastasis assays

All animal experiments were approved by the Animal Care and Use Committee of Jinan Central Hospital affiliated to Shandong University. BALB/c athymic nude mice (4‐6 weeks old) were used for all in vivo studies. To induce ectopic tumour, 2 × 10^6^ cells suspended in 100 mL of medium were subcutaneously injected into right flank of the mice (n = 6). Tumour size was measured every 3 days, and the volume was determined as follows: length × width 2/2. To ascertain the role of α5‐nAChR in tumour metastasis, 2 × 10^6^ cells suspending in 100 μL medium were intravenously injected into the lateral tail vein of nude mice. All mice were killed by cervical vertebra dislocation. The tumour xenografts and the lung were collected and analysed by immunohistochemistry.

### 2.4 Immunohistochemistry

Tissue sections (4 µm thickness) were analysed and detected by immunohistochemical staining using the streptavidin peroxidase method (S‐P method). The sections were deparaffinized, rehydrated with decreasing ethanol concentrations and then immersed in 3% H_2_O_2_ for 10 minutes. Subsequently, the sections were incubated with primary antibodies [anti‐α5‐nAChR mAb (1:400; Abcam), anti‐Jab1 mAb (1:50; Santa), p‐Stat3 (1:50; CST) and N‐cadherin (1:2000; Proteintech Group, Inc)] at 37°C in humidified chambers for 2 hours. After washing with PBS, the sections were incubated with a biotinylated secondary antibody (1:2000, Maixin Bio) for 30 minutes at 37°C. Finally, the sections were visualized by incubation with a 3,3‐diaminobenzidine solution.^14^ The nucleus was counterstained with haematoxylin. Negative controls were performed by omitting the primary antibodies in all cases. The stained tumour cells were rated as follows: negative expression, no positive staining; weak expression, ≤10% of the positively stained cells; and moderate and strong expression, >10% of the positively stained cells. Weak expression was rated as negative, and moderate and strong expressions were rated as positive.

### 2.5 siRNA interference and lentiviral transfection

siRNAs for CHRNA5 (encoding α5‐nAChR), Jab1, Stat3 and the negative control were obtained from GenePharma. Transient transfection of A549 and H1299 cells was performed using the Oligofectamine 2000 (Invitrogen) protocol and 5‐nmol siRNAs in RPMI supplemented with 10% FBS and no penicillin or streptomycin as described previously.^18^ Human CHRNA5 cDNA was subcloned into a pGV‐puro lentiviral vector containing the puromycin resistance to establish stable A549 and H1299 (Genechem) cell lines to induce α5‐nAChR overexpression (marked as α5‐nAChR^+^). Stable A549 and H1299 cells were selected by incubating cells in medium supplemented with puromycin for 48 hours after transfection.

### 2.6 Western blot analysis

Cell monolayers were lysed in RIPA buffer as previously described.^28^ The primary antibodies used in this study included α5‐nAChR (1:800; Genetex), anti‐Jab1 (1:200; Santa), anti‐p‐Stat3 (1:1000; Abcam), anti‐p‐Stat3 (1:1000; Cell Signaling Technology), N‐cadherin (1:2000; Genetex), Vimentin (Proteintech Group, Inc) and anti‐GAPDH (1:2500; Proteintech Group, Inc). GAPDH served as an internal standard.

### 2.7 Wound healing assay

Monolayers of A549 or H1299 cells in the 6‐well plates were wounded by scratching the surface. The wells were rinsed three times with phosphate‐buffered saline (PBS) and incubated at 37°C for 48 hours. The movements of A549 or H1299 cells in the scratched area were photographed by using an Olympus CKX41 microscope. The healing width was calculated from 0 to 48 hours and normalized to the control group.

### 2.8 Cell invasion assay

The invasion assay was performed using a 24‐well Transwell chamber (Corning, USA). A549 or H1299 cells were seeded at a density of 1 × 10^5^ cells into the upper chamber with an 8 mm pore size insert (Corning, USA). The wells of the plate were filled with 600 mL of RPMI1640 supplemented with 20% FBS. A549 or H1299 cells on the upper side of the membrane were removed using clean swabs, and cells on the underside were viewed and counted after incubation for 48 hours at 37°C. A549 or H1299 cell invasion was quantified using an inverted‐contrast microscope.

### 2.9 Statistical analysis

The data are presented as the means ± SDs and were analysed using SPSS v22.0 (SPSS). Student's *t* test was used to assess the significance of the differences between groups. Spearman's correlation analysis was used to analyse the association between α5‐nAChR and Jab1 proteins in NSCLC. The differences were considered significant if *P* was <.05.

## 3 RESULTS

### 3.1 CHRNA5 and Jab1 (COSP5) expression and poor prognosis are correlated in NSCLC patients

**Figure 1:**
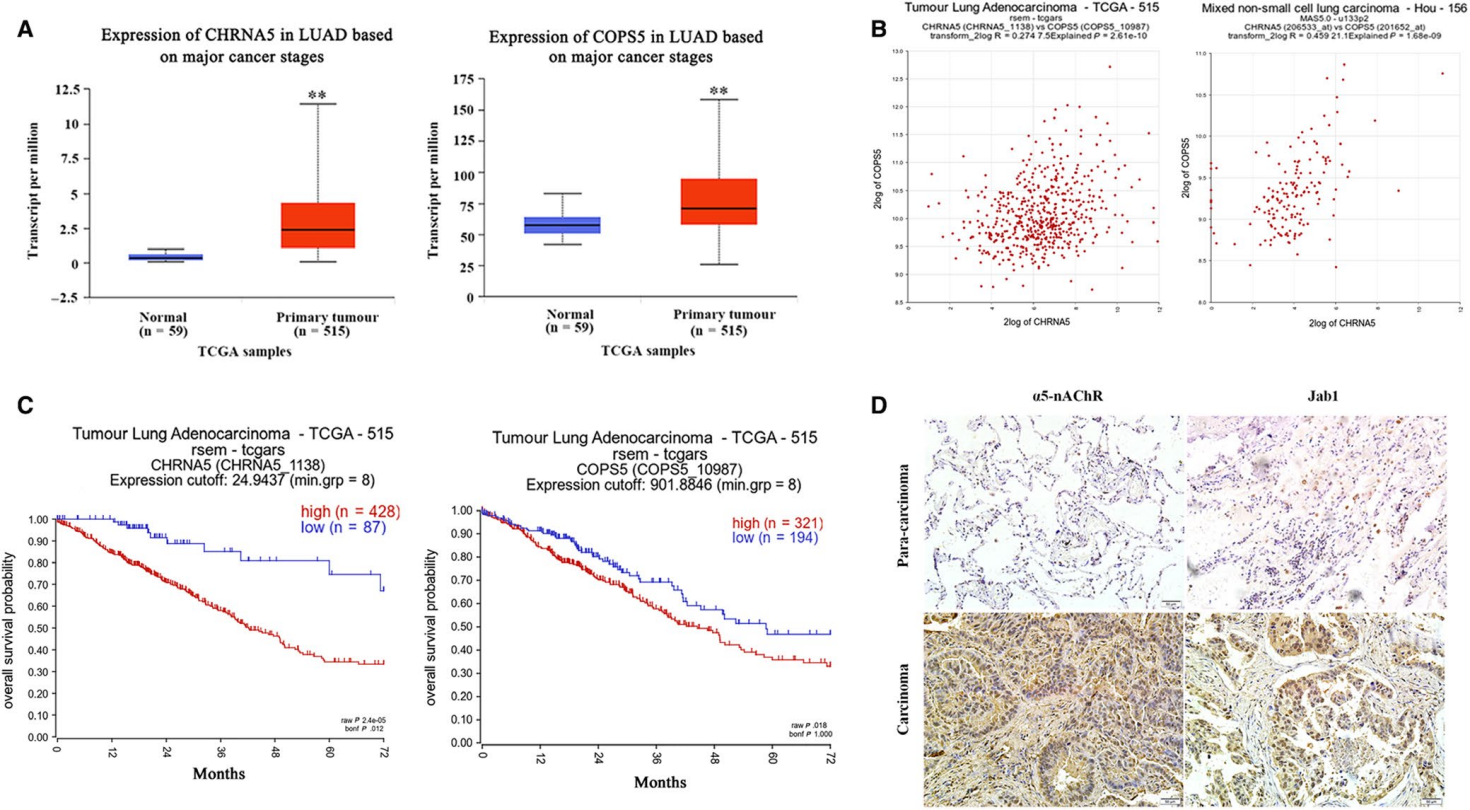
CHRNA5 and Jab1(COPS5) expressions are correlated in lung cancer. A, Data from the Ualcan database show the expression patterns of CHRNA5 and Jab1(COPS5) in NSCLC and normal tissues. B, Correction between the expression of CHRNA5 and Jab1(COPS5) in LUAD, data from R2 online database. C, The association of CHRNA5 and Jab1(COPS5) expression with clinical outcomes in LUAD by R2: Kaplan Meier Scanner (Pro). D, α5‐nAChR and Jab1 expressions in human NSCLC tissues (carcinoma *vs* para‐carcinoma 200×). Scare bar, 50 µm

CHRNA5 and Jab1 (COSP5) expression was increased in the TCGA LUAD subset (n = 515) compared to that observed in the control group using the Ualcan online database (http://ualcan.path.uab.edu/index.html) (Figure 1A). The R2 online database (http://r2.amc.nl) was used to analyse the correlation between CHRNA5 and Jab1 (COSP5) expression in the TCGA LUAD (n = 515) and NSCLC Hou subsets (n = 156). The data showed that CHRNA5 and Jab1 (COSP5) expressions were positively correlated (Figure 1B). The survival analysis using TCGA LUAD subset from R2: Kaplan Meier Scanner (Pro) (https://hgserver1.amc.nl/cgi-bin/r2/main.cgi) showed that high CHRNA5 or Jab1 (COSP5) expression correlated with poor prognosis (Figure 1C). Furthermore, CHRNA5 and Jab1 (COSP5) levels are elevated accompanied by poor prognosis only in smokers and not in non‐smokers (Figure S1).

We previously reported that CHRNA5 expression is significantly associated with overall survival.^18^ In this study, we examined α5‐nAChR and Jab1 expression in 18 NSCLC and 18 para‐carcinoma tissue samples via immunohistochemistry analysis. The results showed that α5‐nAChR was overexpressed in lung cancer tissue (72.2%, 13/18). Consistently, the positive rate of Jab1 in the lung cancer tissue samples (66.7%, 12/18) was higher than that observed in the para‐carcinoma tissue samples (Figure 1D, Table 1). There was a correlation between the expression of α5‐nAChR and that of Jab1 in lung cancer. These data suggest that α5‐nAChR and Jab1 expressions are potential prognostic biomarkers in lung cancer.

**Table 1:** Correlation between α5‐nAChR and Jab1 expression in lung cancer patients

Clinical pathology	Case (n = 18)	Jab1		*P*	α5‐nAChR		*P*
		Negative (n = 6)	Positive (n = 12)		Negative (n = 5)	Positive (n = 13)	
Sex							
Male	8	1	7	>.05	3	5	>.05
Female	10	5	5		2	8	
Age (y)							
≤60	10	3	7	>.05	4	6	>.05
>60	8	3	5		1	7	
TMN stage							
I‐III	10	6	4	.013	5	5	.036
VI	8	0	8		0	8	

### 3.2 α5‐nAChR and Jab1 expressions are correlated in NSCLC tumour xenografts

**Figure 2:**
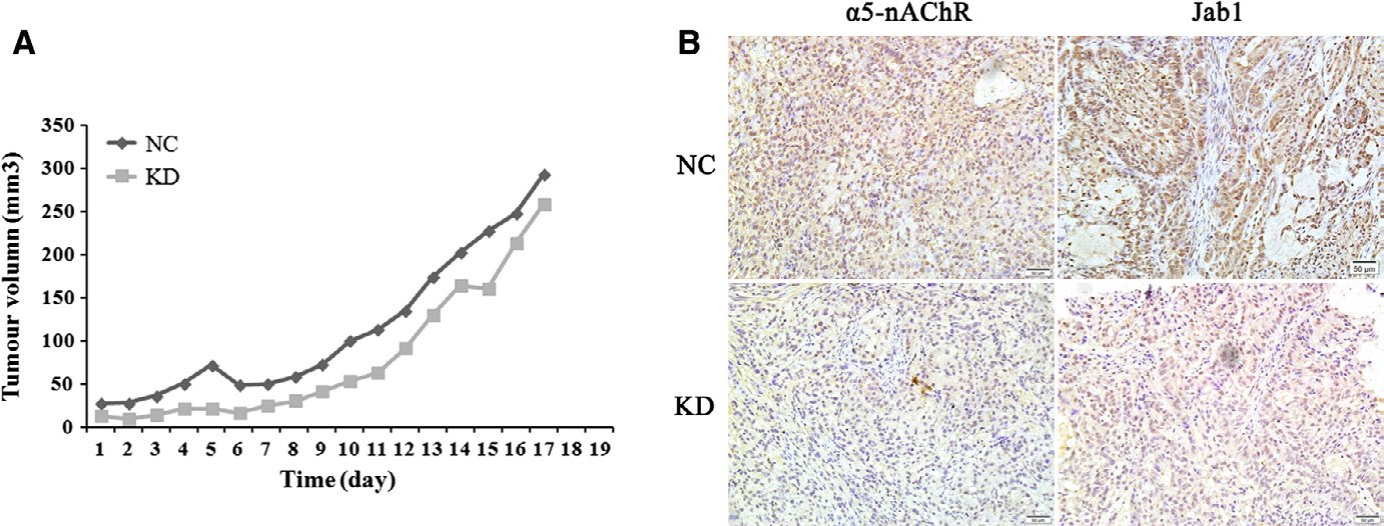
α5‐nAChR and Jab1 expressions in NSCLC tumour xenografts. A, Representative images of harvested tumours (top) and the corresponding tumour growth curves (bottom) are shown. B, The expression of α5‐nAChR and Jab1 in α5‐nAChR knockdown xenografts (KD) and control cells' xenografts (NC) was determined by immunohistochemistry, 200×, scare bar, 50 µm

As α5‐nAChR expression was observed to be associated with Jab1 expression in lung cancer patient samples, we further assessed α5‐nAChR and Jab1 expressions in NSCLC xenograft tissues. As we previously reported,^17^ the tumour volumes of α5‐nAChR knockdown xenografts (KD) were smaller than those of the xenografts derived from control cells (NC) (Figure 2A). The immunohistochemistry assay showed that the expression of α5‐nAChR and Jab1 was higher in the NC group compared to that observed in the sh‐CHRNA5 tumour group (Figure 2B). These results suggested that α5‐nAChR is associated with Jab1 expression in NSCLC carcinogenesis.

### 3.3 α5‐nAChR expression mediates that of Stat3, Jab1, N‐cadherin and Vimentin in A549 and H1299 cells

**Figure 3:**
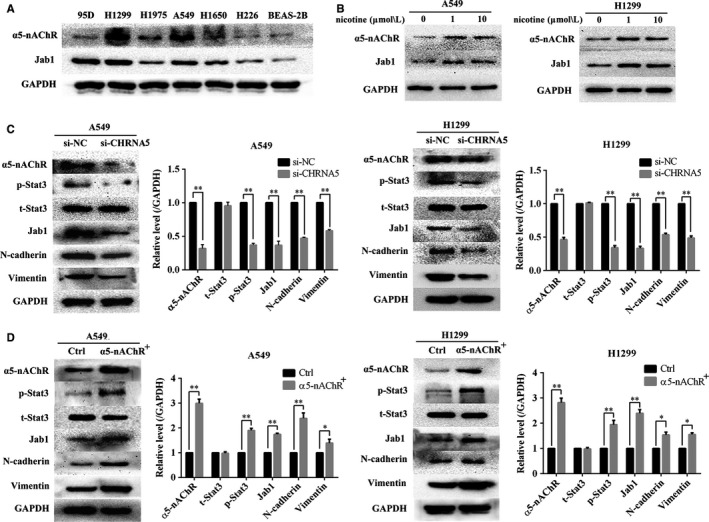
α5‐nAChR expression mediates Stat3, Jab1, N‐cadherin and Vimentin expression in vitro. A, Expression of α5‐nAChR and Jab1 in six NSCLC cell lines (95D, H1299, H1975, A549, H1650 and H226) and the normal epithelial lung cell line (BEAS‐2B). B, The expression of α5‐nAChR and Jab1 was increased by nicotine stimulation in A549 and H1299 cells. C, α5‐nAChR silencing down‐regulated p‐Stat3, Jab1, N‐cadherin and Vimentin in A549 cells and H1299 cells. **P < .01, *P < .05, si‐NC vs si‐CHRNA5. D, Overexpression of α5‐nAChR up‐regulated p‐Stat3, Jab1, N‐cadherin and Vimentin levels in A549 cells and H1299 cells. **P < .01, *P < .05, Ctrl vs α5‐nAChR^+^

Based on the observed correlation between α5‐nAChR and Jab1 expression in vivo, we further explored the association between α5‐nAChR and Jab1 in the NSCLC cells. The expression of α5‐nAChR and Jab1 was detected in six NSCLC cell lines and the normal epithelial lung cell line (BEAS‐2B). Compared to BEAS‐2B cells, α5‐nAChR and Jab1 expression was higher in NSCLC cells (Figure 3A). For further functional experiments, A549 and H1299 cell lines were assayed due to their positive correlation with respect to α5‐nAChR and Jab1 expression. In our previous study, nicotine played a role in a time‐dependent and concentration‐dependent manner. The optimal concentration and treatment time of nicotine on A549 and H1299 cell proliferation are both 1 μM for 16 hours.^28^ The expression of α5‐nAChR and Jab1 expressions was detected in A549 and H1299 cell lines treated with 1 and 10 µM nicotine for 16 hours, and the results showed that α5‐nAChR and Jab1 exhibited increased expression at the protein level compared to that observed in the control (Figure 3B).

Subsequently, we assessed whether there was a link between α5‐nAChR and Stat3‐Jab1 on EMT in NSCLC. Negative control and CHRNA5 RNA interference fragments were transfected into A549 and H1299 cells. The silencing of α5‐nAChR down‐regulated p‐Stat3, Jab1, N‐cadherin and Vimentin expression (*P* < .05) (Figure 3C). Furthermore, the overexpression of α5‐nAChR up‐regulated the levels of p‐Stat3, Jab1, N‐cadherin and Vimentin (*P* < .05) (Figure 3D). No change in the expression of total Stat3 was observed in any of the groups. These results suggest that α5‐nAChR can regulate the expression of p‐Stat3, Jab1 and EMT markers.

### 3.4 α5‐nAChR/Stat3 signalling mediates Jab1 expression in A549 and H1299 cells

**Figure 4:**
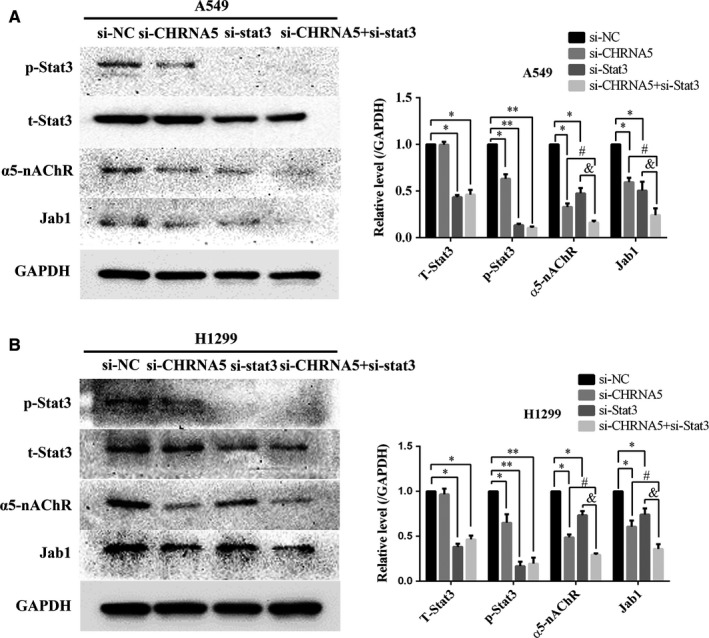
α5‐nAChR and/or Stat3 silencing down‐regulates Jab1 expression. A, Jab1 expression was inhibited after silencing α5‐nAChR and/or Stat3 expression in A549 cells. B, Jab1 expression was inhibited after silencing of α5‐nAChR and/or Stat3 expression in H1299 cells. *P < .05, **P < .01, si‐NC vs si‐gene; ^#^ *P* < .05, si‐CHRNA5 vs si‐CHRNA5 + si‐Stat3; ^&^ *P* < .05, si‐Stat3 vs si‐CHRNA5 + si‐Stat3

We cultured A549 and H1299 cells and transfected them with si‐NC, si‐CHRNA5, si‐Stat3 or si‐CHRNA5 + si‐Stat3 fragments to study the correlation between α5‐nAChR/Stat3 and Jab1. As show in Figure 4A, silencing of α5‐nAChR decreased the levels of p‐Stat3 and Jab1 (*P* < .05), which is generally consistent with the results of our previous study.^18^ Furthermore, silencing Stat3 decreased the levels of α5‐nAChR and Jab1 (*P* < .05). It is worth noting that compared to si‐CHRNA5 cells and si‐Stat3 cells, the expression of α5‐nAChR and Jab1 was lower in si‐CHRNA5 + si‐Stat3 cells (*P* < .05). Similar results were also observed in the H1299 cell line (Figure 4B). These results are consistent with those of our former study showing that there is a feedback loop between α5‐nAChR and Stat3, and also demonstrate that α5‐nAChR/Stat3 signalling mediates Jab1 expression in NSCLC cells.

### 3.5 α5‐nAChR and Jab1 regulate lung cancer cell migration and invasion

**Figure 5:**
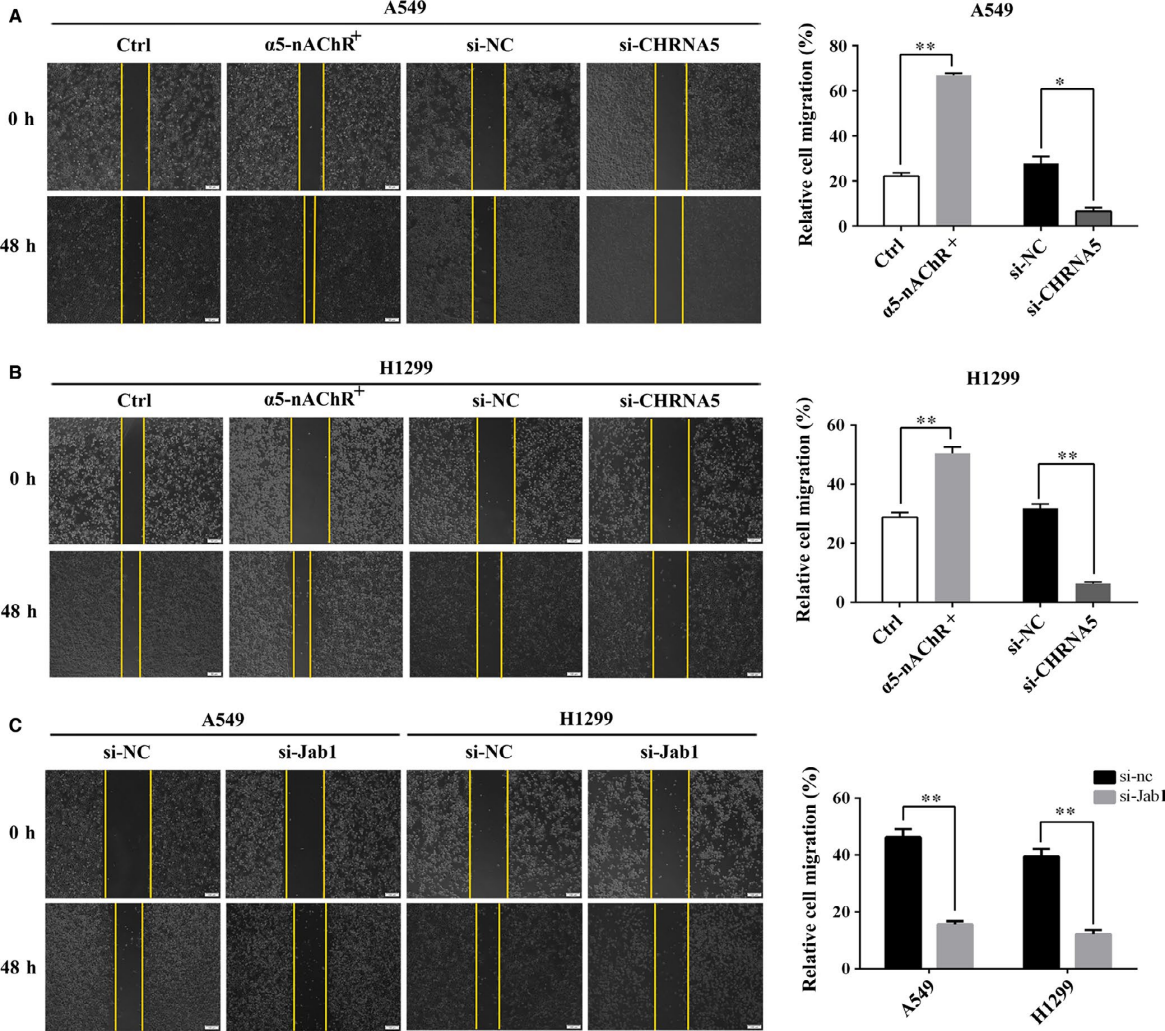
Effects of α5‐nAChR and Jab1 on lung cancer cell migration. A, α5‐nAChR overexpression and down‐regulation enhanced and inhibited the migration of A549 cells, respectively. Wound closure percentages are shown in the right panel. **P < .01, *P < .05, Ctrl vs α5‐nAChR, si‐NC vs si‐CHRNA5. B, α5‐nAChR overexpression and down‐regulation enhanced and inhibited the migration of H1299 cells, respectively. Wound closure percentages are shown in the right panel. **P < .01, Ctrl vs α5‐nAChR, si‐NC vs si‐CHRNA5. C, Jab1 silencing inhibited the migration of A549 and H1299 cells. Wound closure percentages are shown in the right panel. **P < .01, si‐NC vs si‐Jab1

Migration and invasion are closely associated with cancer cell metastasis. To evaluate the effects of α5‐nAChR and Jab1 on the migration and invasion of NSCLC cells, wound healing and transwell assays were performed. The wound healing assay results showed that increased α5‐nAChR expression in A549 cells was associated with a significantly faster wound closure, while silencing α5‐nAChR by si‐CHRNA5 resulted in slower wound healing compared to that observed in the control (Figure 5A). These assays were performed using H1299 cells (Figure 5B). In addition, we transfected cells with si‐Jab1 to study the role of Jab1 in cell migration. Compared to the control cells, si‐Jab1‐transfected A549 and H1299 cells migrated more slowly to repair the wounds (Figure 5C).

**Figure 6:**
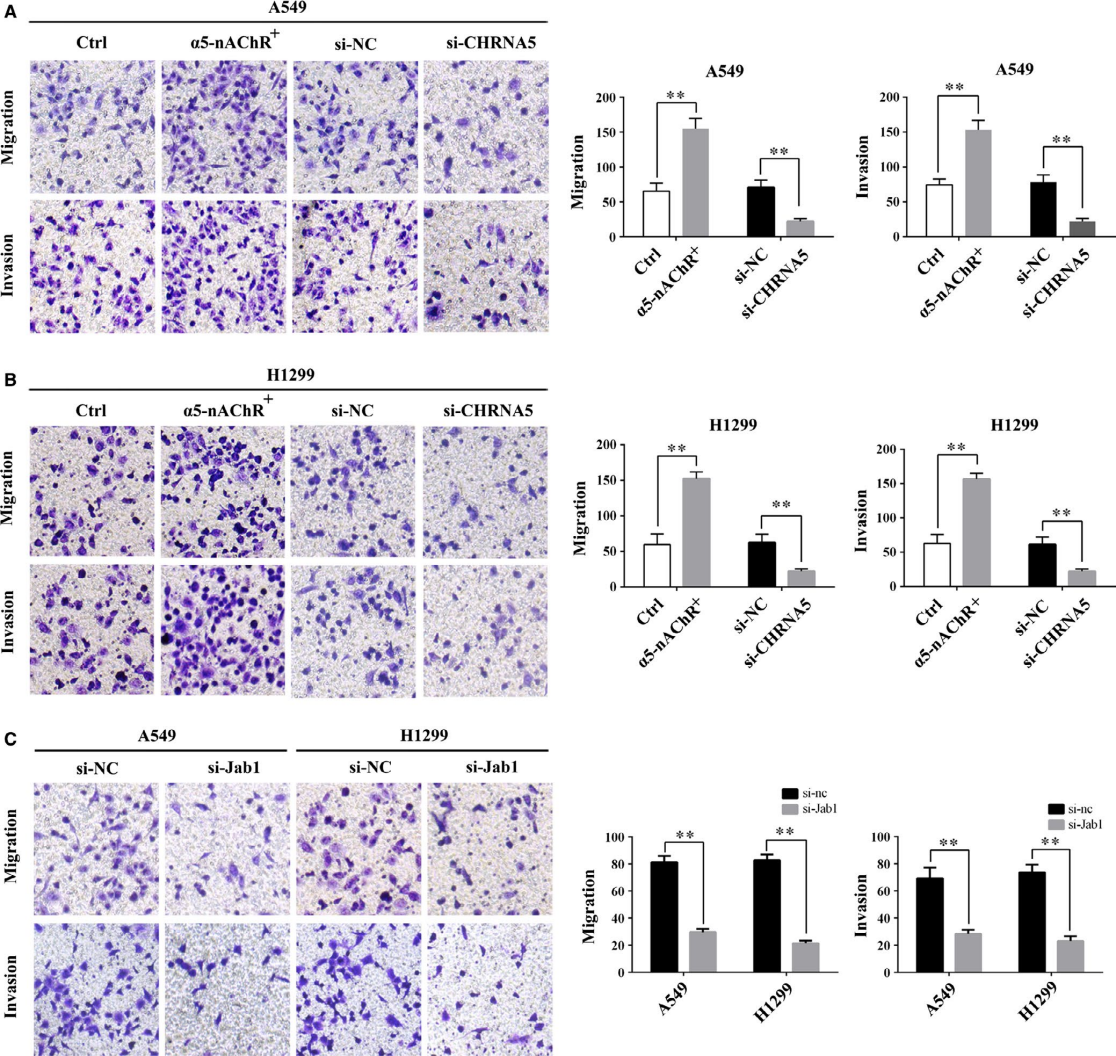
Effects of α5‐nAChR and Jab1 on lung cancer cell invasion. A, α5‐nAChR overexpression and down‐regulation enhanced and inhibited the invasion of A549 cells, respectively. The quantitative results are shown in the right panel. **P < .01, Ctrl vs α5‐nAChR, si‐NC vs si‐CHRNA5. B, α5‐nAChR overexpression and down‐regulation enhanced and inhibited the invasion of H1299 cells, respectively. The quantitative results are shown in the right panel. **P < .01, Ctrl vs α5‐nAChR, si‐NC vs si‐CHRNA5. C, Jab1 silencing inhibited the invasion of A549 and H1299 cells. The quantitative results are shown in the right panel. **P < .01, si‐NC vs si‐Jab1

We performed transwell assays to further verify the role of α5‐nAChR and Jab1 in regulating cell invasion and migration. The results of transwell assays ± Matrigel demonstrated that the overexpression of α5‐nAChR increased A549 cell migration and invasion, whereas α5‐nAChR silencing significantly decreased cell migration and invasion (Figure 6A). Similar results were observed using H1299 cells (Figure 6B). si‐Jab1‐treated cells exhibited decreased migration and invasion compared to the control cells (Figure 6C). These results suggest that α5‐nAChR and Jab1 mediate NSCLC cell migration and invasion in vitro. In addition, to evaluate whether α5‐nAChR expression mediates distant metastasis in vivo, mice were implanted with α5‐nAChR^+^/A549 and corresponding control cells via the tail vein, respectively. At day 36, haematoxylin‐eosin (HE) staining showed that more metastatic foci were observed in the lungs of α5‐nAChR^+^/A549‐treated mice (Figure S2), which confirmed that α5‐nAChR involved in lung cancer metastasis.

## 4 DISCUSSION

The results of our previous study showed that nicotine‐induced activation of JAK2/Stat3 signalling was inhibited by the silencing of α5‐nAChR. ChIP assays confirmed that the CHRNA5 promoter contains Stat3 binding sites. There is a feedback loop between α5‐nAChR and Stat3 that contributes to the nicotine‐induced tumour cell proliferation.^18^ α5‐nAChR modulates lung tumour cells invasion and migration, promoting tumour metastasis.^19^ P‐Stat3 regulates the progression of nasopharyngeal and breast cancer by activating Jab1.^23^, ^24^ JAK/Stat3^29^ and Jab1^25^ are implicated in the mechanisms of EMT in different types of human cancer. Data have shown that phosphorylation of Stat3 on Tyr705, not Ser727, drives a transcriptional program that converts an epithelial morphology to a migratory mesenchymal one. In the present study, we demonstrated that α5‐nAChR mediates NSCLC cell EMT and metastasis via the Stat3/Jab1 axis. The level of α5‐nAChR was correlated with Jab1 in lung cancer in vivo. The results of subsequent assays revealed that the α5‐nAChR/Stat3 signalling mediates Jab1 expression in A549 and H1299 cells. Furthermore, Vimentin and N‐cadherin, which function as EMT markers, were regulated by the α5‐nAChR/Stat3/Jab1 axis and contributed to tumour invasion and migration. In addition, changes in α5‐nAChR and Jab1 levels significantly altered NSCLC cell migration and invasion. These findings reveal that α5‐nAChR mediates p‐Stat3, Jab1, Vimentin and N‐cadherin expression and play an important role in the EMT and metastasis of NSCLC.

The role of nAChRs has been intensely studied in the growth, angiogenesis and metastasis of lung cancer.^13^, ^30^ The reason for this trend is due to GWAS studies showing genetic variations in α5‐α3‐β4 nAChR cluster (CHRNA5‐CHRNA3‐CHRNB4) associated with increased risk of death from lung cancer, COPD and tobacco‐related cancers.^14^, ^31^ Conventionally, the α7‐nAChR is thought to mediate the proliferative, pro‐angiogenic and pro‐metastatic activity of nicotine in lung cancer.^32^ Other genomic variances near CHRNA2 gene were correlated to increased overall risk for developing lung cancer.^33^ Such observations underscore the role for different nAchR subunit in the development and progression of lung cancer. However, the mechanism of various nAchR subunit in lung cancer is far to know.^34^ It is well known that nAChRs function as ligand‐gated cationic channels, and their activation by nicotine promotes an influx of Ca^2+^ and activation of voltage‐gated Ca^2+^ channels.^35^ Subsequently, various signalling pathways are activated, such as Wnt/‐catenin, Ras/Raf/MEK/ERK, PI3K/Akt and JAK2/Stat3, leading to the promotion of tumour cell proliferation, EMT and migration.^36^, ^37^, ^38^, ^39^, ^40^ Nicotine and its derivatives such as NNK and NNN, can not only activate nAChRs, studies have also shown that lung cancers synthesize acetylcholine (ACh) and cytokines, which acted as autocrine growth factors that stimulate nAChRs in lung cancer despite a lack of exogenous nicotine.^41^, ^42^ EMT is a key initiating event in the metastatic cascades of various cancers.^43^, ^44^, ^45^ The molecular mechanism of α5‐nAChR in lung cancer EMT is far from clear. In this study, we showed that the expression of α5‐nAChR mediates Stat3/Jab1 signalling and promotes EMT by up‐regulating N‐cadherin and Vimentin expression. Furthermore, α5‐nAChR activation promoted tumour invasion and metastasis in A549 and H1299 cells, validating our in vivo observations. These results suggest that the expression of α5‐nAChR promotes EMT and metastasis by regulating the Stat3/Jab1 signalling pathway in NSCLC.

Jab1 is a tumour oncogene in a variety of human cancers^46^, ^47^ and has been shown to be a key molecule in nicotine‐induced lung cancer.^48^, ^49^ Remarkably, Csn5/Jab1 mediates stabilization of PD‐L1, which is crucial for breast cancer cells to escape immune surveillance via PD‐L1/PD‐1 interaction. Immune checkpoint‐blockade treatments targeting PD‐1/PD‐L1 have revolutionized various cancers therapy. Thus, Jab1 represents a potential target for the treatment of inflammatory‐related cancers. In this study, α5‐nAChR expression was shown to be correlated with that of Jab1 in lung cancer in vivo. α5‐nAChR/Stat3 signalling mediates Jab1 and EMT molecules in A549 and H1299 cells. The inhibition of α5‐nAChR or Jab1 inhibited the migration and invasion of NSCLC. Our results demonstrated that α5‐nAChR/Jab1 signalling may be an important mechanism responsible for promoting the EMT, invasion and migration of NSCLC. Thus, targeting α5‐nAChR/Jab1 signalling in cancer cells may be a potential strategy for cancer treatment via mediating PD‐1/PD‐L1 signalling. Based on the results of this study of α5‐nAChR/Jab1 expression in the EMT and metastasis of NSCLC, further studies will be performed to assay the role of the α5‐nAChR/Jab1/PD‐L1 axis in lung cancer immunotherapy.

In summary, we demonstrated that α5‐nAChR mediates NSCLC EMT, migration and invasion via Stat3/Jab1. These findings provide new insights into the possible molecular mechanisms by which α5‐nAChR and Jab1 mediate lung cancer metastasis. Acetylcholine and its analogue bind to α5‐nAChR on the cell surface and activate p‐Stat3, which subsequently activates the expression of Jab1 to promote tumour cell EMT and metastasis (Figure S3).

As Jab1 is important for cancer cells to escape immune surveillance by mediating PD‐L1/PD‐1 interactions, the association between the α5‐nAChR/Jab1 axis and PD‐L1 requires further study. Thus, components of the α5‐nAChR/Jab1/PD‐L1 axis may be useful as potential targets for NSCLC diagnosis and immunotherapy in the future.

## CONFLICT OF INTEREST

The authors confirm that there are no conflicts of interest.

## AUTHOR CONTRIBUTIONS

XC and XM wrote the first draft of the manuscript. HS and XM contributed to the conception and design of the research. XC, YJ, YZ and DZ contributed to the experiment and interpretation of data. XC, HS and XM revised the paper. All authors approved the final version of the manuscript.

## Data Availability

The data used to support the findings of this study are available from the corresponding author upon reasonable request.
