# Supplementary figures and images for: α5‐nAChR contributes to epithelial‐mesenchymal transition and metastasis by regulating Jab1/Csn5 signalling in lung cancer
Source: J Cell Mol Med. 2020 Jan 13;24(4):2497–506. doi: 10.1111/jcmm.14941 (PMC7028847; doi:10.1111/jcmm.14941)

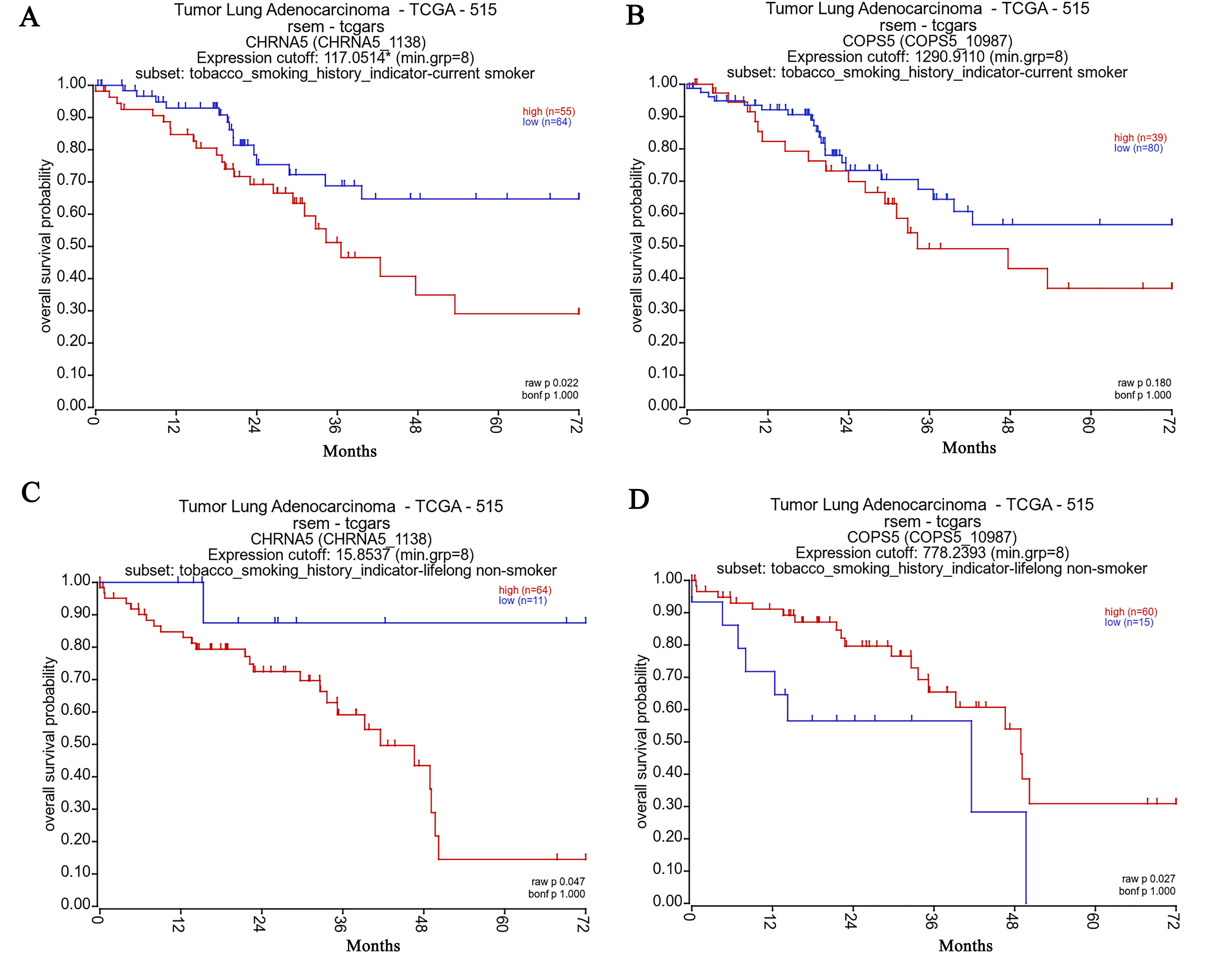

Supplement: Supplementary file 1 [file JCMM-24-2497-s001.tif]

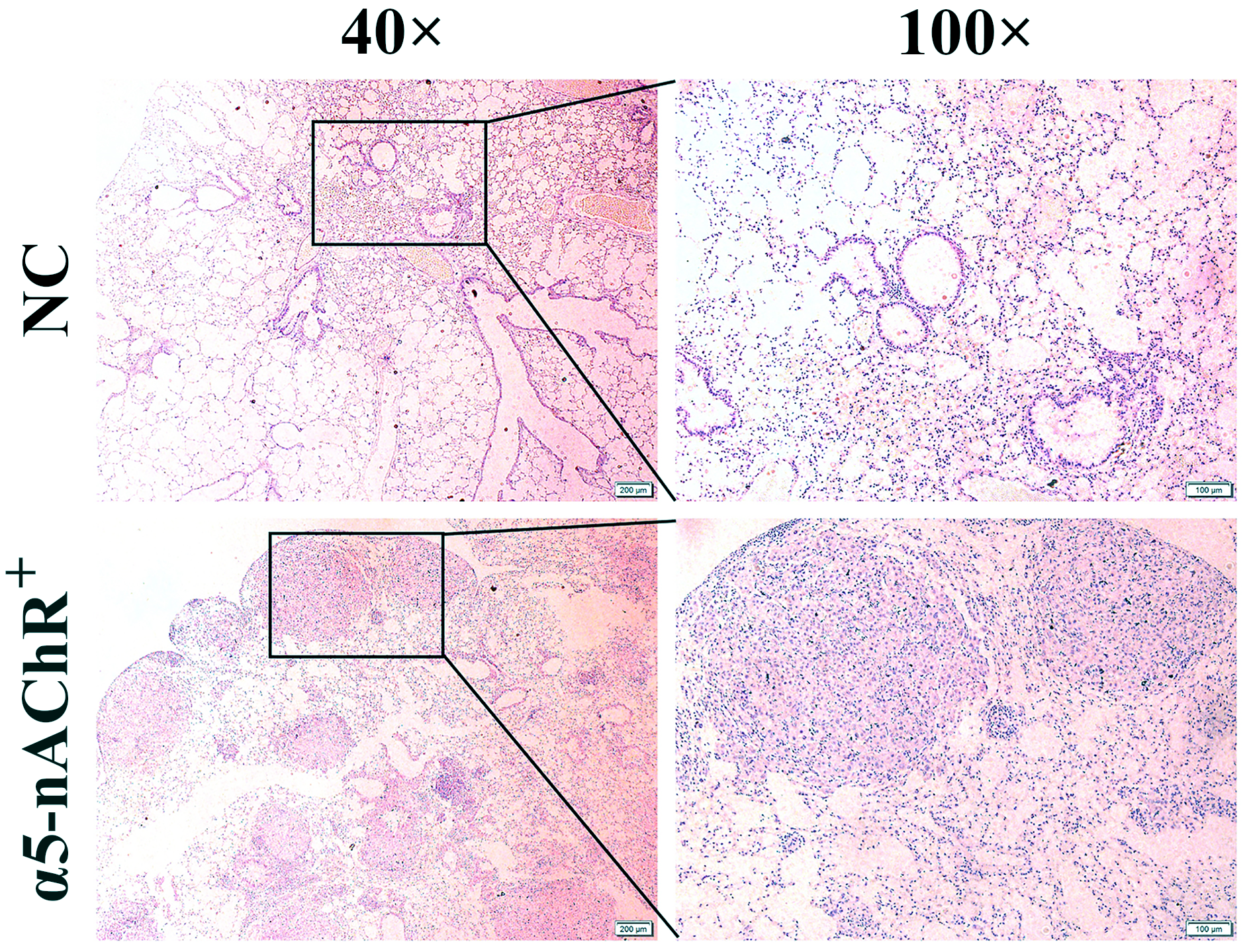

Supplement: Supplementary file 2 [file JCMM-24-2497-s002.tif]

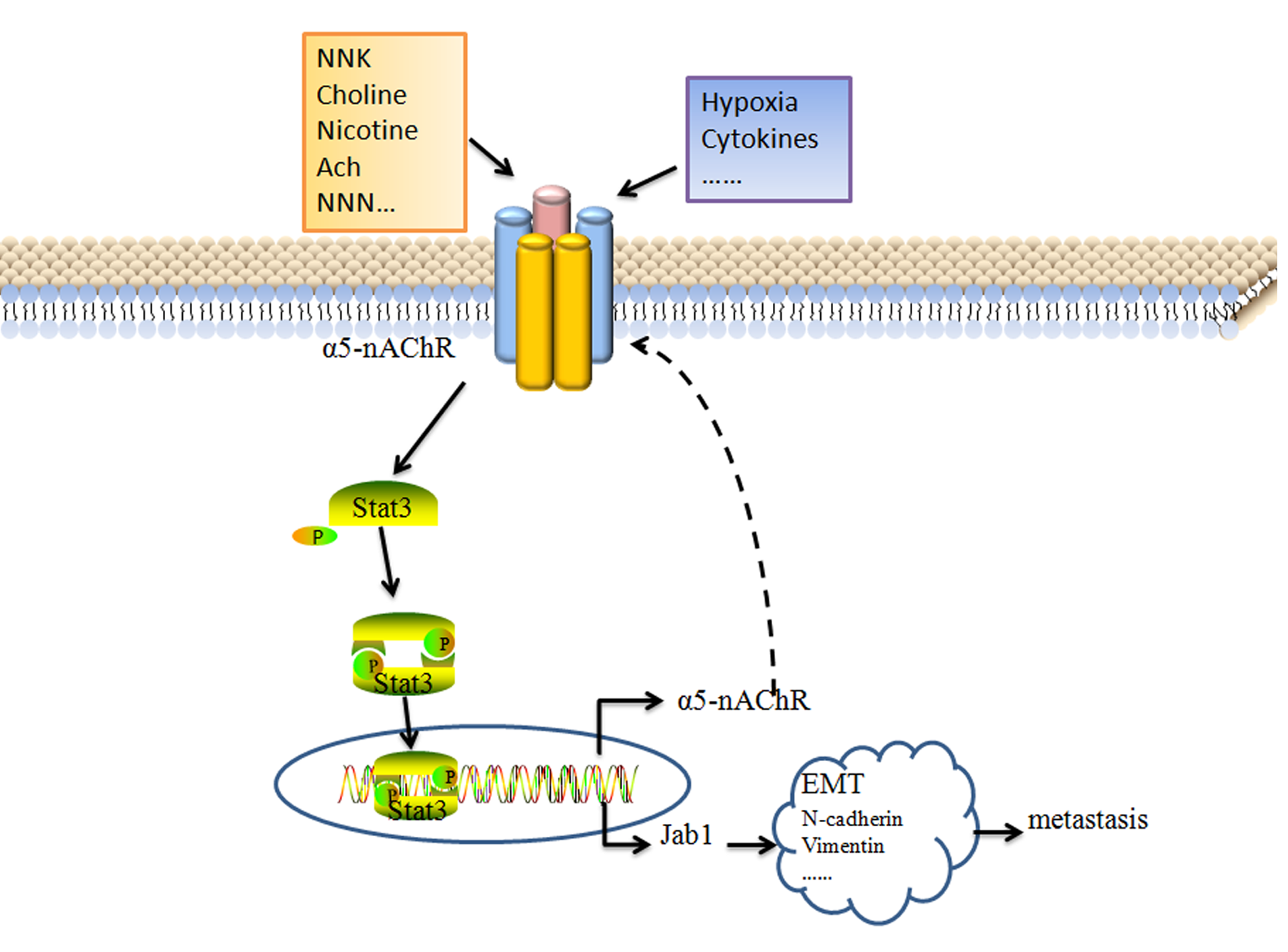

Supplement: Supplementary file 3 [file JCMM-24-2497-s003.tif]
