# Supplementary material for: α5‐nAChR contributes to epithelial‐mesenchymal transition and metastasis by regulating Jab1/Csn5 signalling in lung cancer
Source: J Cell Mol Med. 2020 Jan 13;24(4):2497–506. doi: 10.1111/jcmm.14941 (PMC7028847; doi:10.1111/jcmm.14941)
Supplement: Supplementary file 4 [file JCMM-24-2497-s004.docx]

Review

Figure S1 Kaplan-Meier curves for overall survival of CHRNA5 and COPS5 in smokers or non-smokers TCGA LUAD patients

Figure S2 α5-nAChR expression was associated with metastasis in vivo

Figure S3 Proposed signaling cascades of α5-nAChR, Stat3and Jab1 in lung cancer EMT and metastasis
